# Recommendations for Implementing Innovative Technologies to Control *Aedes aegypti*: Population Suppression Using a Combination of the Incompatible and Sterile Insect Techniques (IIT-SIT), Based on the Mexican Experience/Initiative

**DOI:** 10.3390/insects15120987

**Published:** 2024-12-12

**Authors:** Abdiel Martín-Park, Yamili Contreras-Perera, Azael Che-Mendoza, Silvia Pérez-Carrillo, Norma Pavía-Ruz, Josué Villegas-Chim, Emilio Trujillo-Peña, Wilberth Bibiano-Marín, Anuar Medina-Barreiro, Gabriela González-Olvera, Juan Navarrete-Carballo, Henry Puerta-Guardo, Guadalupe Ayora-Talavera, Hugo Delfín-González, Jorge Palacio-Vargas, Fabián Correa-Morales, Haroldo Sergio da Silva Bezerra, Giovanni Coelho, Gonzalo Vazquez-Prokopec, Zhiyong Xi, Pablo Manrique-Saide, Héctor Gómez-Dantes

**Affiliations:** 1 Laboratorio para el Control Biológico de *Aedes aegypti* (LCB-UADY), Unidad Colaborativa de Bioensayos Entomológicos, Campus de Ciencias Biológicas y Agropecuarias, Universidad Autónoma de Yucatán, Merida 97100, Mexico; ampark27@gmail.com (A.M.-P.); yamjaz_85@hotmail.com (Y.C.-P.); achemendoza_vectores@hotmail.com (A.C.-M.); silmaggui@gmail.com (S.P.-C.); jovich.etnos@gmail.com (J.V.-C.); emilio_trujillo20@hotmail.com (E.T.-P.); wbm.biol@gmail.com (W.B.-M.); anuar116@hotmail.com (A.M.-B.); gabygzzo@gmail.com (G.G.-O.); jcarbalho85@gmail.com (J.N.-C.); hpuertaguardo@gmail.com (H.P.-G.); gdelfin@correo.uady.mx (H.D.-G.); 2 Institut de Recherche pour le Développement (IRD), MIVEGEC, University of Montpellier, 34394 Montpellier, France; 3 Centro de Investigaciones Regionales, Unidad Biomédicas, Universidad Autónoma de Yucatán, Merida 97000, Mexico; pruz@correo.uady.mx (N.P.-R.); talavera@correo.uady.mx (G.A.-T.); 4 Servicios de Salud de Yucatán, Merida 97000, Mexico; piulg_1985@hotmail.com; 5 Centro Nacional de Programas Preventivos y Control de Enfermedades (CENAPRECE), Secretaría de Salud, Mexico 11410, Mexico; fabiancorrea@msn.com; 6 Pan-American Health Organization/World Health Organization, Washington, DC 20037, USA; bezerrha@paho.org (H.S.d.S.B.); coelhogio@paho.org (G.C.); 7 Department of Environmental Sciences, Emory University, Atlanta, GA 30322, USA; gmvazqu@emory.edu; 8 Department of Microbiology, Genetics, & Immunology, Michigan State University, East Lansing, MI 48824-4320, USA; xizy@msu.edu; 9 Centro de Investigación en Sistemas de Salud, Instituto Nacional de Salud Pública, Cuernavaca 62100, Mexico

**Keywords:** *Wolbachia*, incompatible insect technique (IIT), sterile insect technique (SIT), *Aedes aegypti*, Mexico

## Abstract

The future of *Aedes aegypti* control emphasizes the transition from traditional insecticides toward more sustainable and multisectoral integrated strategies, like using *Wolbachia*-carrying mosquitoes for population suppression or replacement. We reviewed the integration of the successful Mexican initiative, “Mosquitos Buenos”, with the key challenges outlined in the PAHO guidelines for incorporating innovative approaches into vector control programs. These challenges include establishing essential infrastructure, training personnel, managing field operations, and fostering community support. Our experience provides critical evidence to support the strategic National Plan for implementing and integrating IIT-SIT technologies to control *Ae. aegypti* and dengue. Furthermore, this experience serves as a foundation for other countries in the region interested in adopting these technologies. It underscores the importance of strategic planning, multisectoral collaboration, continuous evaluation, and scaling up innovative tools to ensure their long-term effectiveness and sustainability in urban areas where *Aedes* vectors and the diseases they transmit are endemic.

## Simple Summary

The *Wolbachia*-based incompatible insect technique (IIT) and the sterile insect technique (SIT) are effective technological innovations to control *Aedes* mosquitoes and could be implemented as part of integrated, synergistic, and complementary schemes to enhance the efficiency and coverage of traditional control tools. This review presents the implementation of an IIT-SIT strategy for population suppression within an integrated vector management plan operated by the Ministry of Health in Mexico, and assesses its adherence to the different prerequisites, indicators of agreement, concordance, and suggestions proposed in the PAHO guidelines for the adoption of innovations in the control of *Ae. aegypti*. This experience provides essential evidence for the strategic national plan for the implementation and integration of IIT and SIT for the control of *Ae. aegypti* and dengue and should serve as an example to other countries in the region interested in implementing these technologies, since it illustrates the complex nature of adopting and trying to scale up this kind of innovative tool in urban settings where *Aedes* vectors and *Aedes*-borne diseases are endemic.

## 1. Introduction

The future of *Aedes aegypti* control must evolve from a reactive, strongly insecticide-dependent and vertical approach to an integrated and rational combination of preventive and reactive strategies implemented with a multisectoral approach and applied synergistically to target different stages of the mosquito population in time and space. Various innovative *Ae. aegypti* control strategies have been developed in the past decade as a response to current shortcomings in routine vector control [1], including some within the intervention class known as “sterilization agents” [1,2].

*Wolbachia*-carrying mosquitoes represent a novel paradigm which involves massive production and release (‘rear-and-release’) of male and female mosquitoes carrying a *Wolbachia* strain with capacity to reduce the ability to transmit *Aedes*-borne viruses (ABVs) (dengue [DENV], Zika [ZIKV], and chikungunya [CHIKV]) [3]. On the other hand, the *Wolbachia*-mediated population suppression strategy, known as the incompatible insect technique (IIT), involves releasing large numbers of male *Aedes* with *Wolbachia*, which, when mating with a wild-type female, lead to the death of the embryo or the production of non-viable eggs through a process termed cytoplasmic incompatibility (CI) [4,5].

Both approaches to *Wolbachia*-based biocontrol (WBC) of *Ae. aegypti* have transitioned through different levels of implementation, showing promising results after entomological and epidemiological field trials [4,6,7,8,9,10,11]. The use of *w*Mel for *Ae. aegypti* population replacement, led by the World Mosquito Program (WMP), has received significant funding to develop pilot studies for DENV control in 14 countries. The evidence of its public health value has resulted in a positive recommendation by the Vector Control Advisory Group (VCAG) of the World Health Organization (WHO) and the initiation of the development of guidelines for this intervention [12]. *Wolbachia*-mediated population suppression with IIT or IIT combined with the sterile insect technique (SIT) (irradiated mass-reared adults to reduce the risk of releasing fertile females) has followed a much slower process. Nevertheless, various initiatives implementing WBC for population suppression of both *Ae. aegypti* and *Ae. albopictus* have taken place and/or are ongoing in different countries [4,5,10,13,14,15,16,17], and some of this evidence has been presented to VCAG for its consideration [2].

Considering the process of evaluating their entomological efficacy and public health value (i.e., epidemiological impact against target diseases), it would be premature to fully recommend the adoption of these innovative techniques further evidence of their scalability and operational efficacy. Programmatic use of such innovations and their impact in real-life situations is still limited and is expected to be phased in gradually. Vector control programs deciding the adoption of these new technologies will need to make significant adjustments in their structure, organization, focus, procedures, and even their budgets, to be able to deploy these effectively and synergistically as part of an integrated vector management (IVM) approach [18]. Their implementation must be evaluated in the light of local capacities and the integrated use of other control tools [19]. Like all available tools, WBC of *Ae. aegypti* should be used as part of an integrated and synergistic scheme of tools with specific targets and stages of implementation to maximize the combined effects of the interventions [20] and should not be seen as a “silver bullet” that will replace existing interventions, but rather as an advance to improve control programs.

A key challenge for the eventual incorporation of WBC of *Ae. aegypti* and other innovative technologies into institutional vector control programs is their availability and accessibility. Most of the countries affected by ABVs do not currently have the necessary infrastructure, trained personnel, or political and financial support needed, especially in the large urban areas that account for the greatest burden of DENV, ZIKV, and CHIKV. Technical personnel will need to be trained and prepared to ensure production, availability, accessibility, and quality control processes. Additionally, they should be equipped to identify and overcome challenges during the introduction, implementation, and scaling up of IVM when these programs are delivered to communities. Mass production requires specialized facilities to ensure adequate production in addition to monitoring the traits of the released/introduced populations to ensure the continuity and success of the interventions. In terms of volume and frequency, the production capacity required to cover extensive areas is a challenge, not only because of the magnitude of the task but the resources required to carry it out. In addition, significant efforts must be made to educate the population to ensure acceptance and adherence to these new technologies and interventions. The use of new tools involving production and mass release of mosquitoes entails a multifaceted process and a change of paradigm, where instead of visibly “killing” the mosquitoes, we use them as an “autodissemination agent” for biorational vector control.

When deciding whether to adopt new tools for the control of *Ae. aegypti*, it is essential to have a guide for assessing the capacity of local programs for their introduction, implementation, scale-up, monitoring, and impact assessment [21]. The Pan American Health Organization (PAHO) [18] has published two sets of guidelines as part of the roadmap to enhance the control of *Ae. aegypti*, DENV, and urban ABVs in the Americas: (1) *Evaluation of Innovative Strategies for Aedes aegypti Control: Challenges for their Introduction and Impact Assessment* [18] and (2) the *Technical document for the implementation of interventions based on generic operational scenarios for Ae. aegypti control* [22]. These comprehensive documents were produced to provide a review of the broad range of available interventions, including mass release of mosquitoes and WBC of *Ae. aegypti*, and they discuss the scientific and technical elements needed to comprehend the potential advantages and limitations, while suggesting a “checklist” for countries willing to implement such innovations as part of their existing vector control programs in the short, medium, and long term. Moreover, rationalization of relevant operational requirements for introducing any new technologies in vector control programs in the region is also provided, as well as a framework for selecting the best tools, targeted for their most efficient use through risk stratification and scenarios.

In 2016, the Government of the Mexican state of Yucatán (GoY) signed an international collaboration agreement with Michigan State University (MSU) and the Autonomous University of Yucatán (UADY) for the development and application of strategies based on a combination of IIT and SIT [4] to suppress *Ae. aegypti* populations. This approach involved the production and release of mass-reared male *Aedes* mosquitoes infected with *Wolbachia w*AlbB [23] that had been irradiated with X-rays in the pupal stage. Due to cytoplasmic incompatibility (CI), wild-type female *Ae. aegypti* mating with released males produce infertile eggs. Furthermore, any *Wolbachia*-carrying female accidentally released is not able to reproduce, minimizing any undesirable establishment of *Wolbachia* in the wild. The *w*AlbB strain of *Wolbachia* is of relevance for IIT, as it has shown its stable and strong CI in *Ae. aegypti*, with minimal effects on mosquito fitness [23]. Given that *w*AlbB in *Ae. aegypti* does not affect male mating success [24] and shows great stability at high temperatures [25], it has been suggested as a suitable option for population replacement/suppression in warm/tropical climates [7].

The Collaborative Unit for Entomological Bioassays (UCBE) and the Laboratory for Biological Control of *Aedes aegypti* (LCB) at the UADY have been evaluating a series of different control interventions (e.g., house screening, insecticide-treated materials, spatial repellents/emanators, targeted indoor residual spraying and IIT-SIT) for the control of *Ae. aegypti* and ABVs [10,26,27,28,29]. LCB-UADY, by request of the GoY and supported by FOMIX-CONACYT (National Council for Science and Technology) and USAID (United States Agency for International Development), developed the first successful pilot field test evaluating the effectiveness of IIT-SIT against *Ae. aegypti*, within an integrated vector management plan implemented by MoH personnel in Mexico. The report has been shared with the public and scientific community in several forums and published in scientific journals [10,30,31,32]. It was confirmed that releases of sterile male mosquitoes significantly reduced the presence and abundance of *Ae. aegypti* females, particularly inside households with 90% (95% C.I. = 0.01–0.66, *p* = 0.019) fewer *Ae. aegypti* females (including those blood-fed) in the season of greatest abundance, compared with the control site. It was also shown that *Wolbachia*-carrying *Ae. aegypti* males could be mass-produced efficiently and with high quality control at the LCB-UADY, and that their release could be successfully implemented as part of an IVM plan with the participation of the Mexican MoH. Based on these successful and promising results, the LCB-UADY moved forward the collaboration with the MoH of Yucatán and the National Center of Preventive Programs and Diseases Control (CENAPRECE) to provide biological material and technical support (including training) for the implementation, integration, and expansion of WBC techniques as part of institutional *Ae. aegypti* and DENV control programs in Yucatan and other DENV-endemic areas of southern Mexican states. The vision of this Mexican non-profit initiative is to make these technologies accessible; thus, the biological material (Mexican *Aedes* mosquitoes with *Wolbachia*), equipment capacity, and infrastructure are available for vector control programs in Mexico.

In the face of the potential deployment of WBC initiatives against *Ae. aegypti* in Mexico and other countries in the Americas, we present an assessment of the level of agreement and compliance with the use of the combined incompatible and sterile insect techniques (IIT-SIT) in Mexico, according to the PAHO guidelines for the “in-country” adoption of innovations in *Aedes aegypti* control, which list the basic requirements for MoH control programs to incorporate (adopt, adapt, or implement) innovations such as WBC of *Ae. aegypti* into their control interventions [18], and how these are fulfilled and addressed in the case of the implementation of IIT-SIT in Mexico. This experience should serve as an example to other countries in the region interested in implementing WBC and illustrates the complex nature of adopting and trying to scale up this kind of innovative tool in urban settings where ABV diseases are endemic.

## 2. Requirements for the Adoption of the Innovation

Table 1 lists basic requirements that a control program in a country must meet when planning to incorporate WBC for *Ae. aegypti* control into its programmatic interventions, specifically via IIT-SIT [18]. In Mexico, an initial pilot field trial was conducted [10], and WBC of *Ae. aegypti* with IIT-SIT is in the process of being scaled-up as part of an IVM-I*A*M approach by the MoH in urban areas in the state of Yucatán, as part of a national strategic plan for its implementation and integration as part of *Ae. aegypti* and DENV control in Mexico. Specific information, responses, situations, and actions taken and/or to be taken in Mexico after the experience of the first pilot test evaluating IIT-SIT against *Ae. aegypti* within an IVM implemented by the MoH are described throughout the text (see also Tables S1 and S2 for a summary).

### 2.1. Local Experience in the Use of Similar Technologies, Including Pilot Studies

The incompatible insect technique (IIT) for *Aedes* control is a sterile insect-based suppression method that uses releases of male mosquitoes. *Wolbachia* (endosymbiotic bacteria) can cause a type of sterility known as CI, in which mating of an uninfected female and an infected male result in the failure of the embryo to develop. This does not occur (a fertile embryo is produced) when mating when mating involves females infected with the same *Wolbachia* strain [33]. *Wolbachia* is not found naturally in *Ae. aegypti* but can be transinfected in the lab with *Wolbachia* from other insects (*v.gr. w*Mel or *w*AlbB from *Drosophila melanogaster* and *Aedes albopictus*, respectively) [23,34]. Once transinfected, female *Wolbachia*-carrying *Ae. aegypti* pass the bacterium down to all their offspring, generation after generation [35,36]. Mass rearing of stable lines of *Ae. aegypti* with *Wolbachia* mosquitoes allows the production of large numbers of male mosquitoes for release, without the need for further microinjection of eggs.

A recent pilot trial in Mexico used a local line of *Ae. aegypti* with *w*AlbB available at LCB-UADY and produced with IIT-SIT after backcrosses (10 generations) with *Wolbachia*-infected *Ae. aegypti* line WB2 [36]. Mass controlled releases of IIT-SIT males to suppress wild populations of *Ae. aegypti* resulted in a 90% reduction in indoor female mosquitoes. That study, which demonstrated the successful integration of *Aedes*–*Wolbachia* IIT-SIT within an IVM plan implemented by the MoH, is unique not only in Mexico but also throughout Latin America [10].

The Singapore *Wolbachia* program is one of the world leaders in the programmatic and institutionalized use of IIT *Wolbachia*–*Aedes* suppression technology for the control of *Ae. aegypti* and DENV. Results have shown that releases of male *Ae. aegypti* with *w*AlbB-infected achieved >90% reductions in the *Ae. aegypti* mosquito populations. Interventions demonstrated an efficacy of up to 77% (121/156, 95% C.I. = 75.81–78.58), with a lower likelihood of DENV cases in release areas [17,37]. The WMP, formerly known as the Eliminate Dengue Program, is a non-profit consortium led by Monash University that is working to develop a *Wolbachia*-based replacement population strategy to control *Ae. aegypti* virus transmission [6]. Since 2011, the WMP has conducted pilot trials in three continents (America, Asia, and Oceania) and 14 countries including Mexico, originally with the *w*Mel strain [38]. In 2021, the results of a three-year randomized controlled study in Yogyakarta (Indonesia) found that deployment of *Wolbachia* reduced DENV incidence by 77% and hospitalizations by 86% [39]. Mexico had successfully managed SIT-based control programs against agricultural and livestock pests [40], like the eradication of the New World screwworm (*Cochliomyia hominivorax*) from North and Central America and the Mediterranean fruit fly (Medfly, *Ceratitis capitata*), with the Moscamed eradication program [41].

### 2.2. Compliance with Regulatory and Legislative Framework for the Use of Biotechnologies in the Health Field (Environmental, Biosafety, Bioethics)

An initial examination must be conducted to identify significant and non-significant human health and environmental impacts related to the proposed activities and to determine whether an environmental assessment is required based on the identified impacts. The pilot trial for IVM integrating *Ae. aegypti* population suppression with IIT-SIT in Yucatán, Mexico was carried out in consensus with the MoH of Yucatan, with the statement of approval required by USAID’s environmental regulations, the MoH of Yucatán, and the Department of the Environment and Natural Resources (SEMARNAT) [36]. The protocol of the pilot project was approved by the Review Board of the Campus of Biological, Veterinary and Agricultural Sciences of UADY (CB-CCBA-I-2019-005). The open-field mosquito releases were approved by the government and the health authorities of Yucatán, and approval (>95%) and informed consent were obtained from members of the local communities [10,31,32].

### 2.3. Availability and Accessibility to Information About the Technology, v.gr. Protocols for Mass Production, Implementation, Etc

LCB-UADY has produced an open-access handbook of the standard operating procedures for the mass production of *Ae. aegypti* with *Wolbachia* and associated control procedures [42], following those established by Michigan State University and Sun Yat-sen University Joint Center of Vector Control for Tropical Diseases in Guangzhou, China [4,43]. Comprehensive reports on the implementation should cover distribution, release, monitoring, and evaluation of efficacy published in open-access peer-reviewed scientific publications [10,30]. Information regarding the transfer of the methodology is fully accessible to national and local MoHs.

### 2.4. Portfolio (Dossier) of Evidence on Safety, Quality, and Efficacy of the Product

The World Health Organization Technical Advisory Group (WHO-TAG) is currently evaluating several innovative approaches and product classes relating to the development of a target product profile (TPP) for *Ae. aegypti*–*Wolbachia* strains for population replacement approaches and population suppression products [44]. Although the WHO has not yet published a reference for the specific case of population suppression with IIT and/or IIT-SIT, there is sufficient evidence of the characteristics of *Ae. aegypti* with *w*AlbB used in Mexico (*w*MID) (Table 2), which are listed below:

### 2.5. Multidisciplinary Scientific Support Group

CENAPRECE from the MoH of Mexico recently created a committee of experts for the evaluation of future implementation of innovative methods of control of *Ae. aegypti*, as part of a plan to strengthen the control of urban arboviruses transmitted by *Aedes*. This multidisciplinary committee is made up of distinguished researchers from different Mexican or international institutions, from academia and government. In addition, each state may have local expert committees to support vector programs, in coordination with corresponding national committees. For example, in Yucatán, the Interinstitutional Council for the Prevention and Control of Mosquito-Transmitted Diseases was established in 2017, as mandated by the Law for the Prevention and Control of Mosquito-Transmitted Diseases in the State of Yucatán (December 2016). A multisectoral operational research committee for the prevention of mosquito-transmitted diseases were also established for the development of strategies, actions, and research contributing to the knowledge and strengthening of prevention and control actions to address mosquito vectors through the active participation of the various research institutions.

UCBE and LCB at UADY are independent academic non-profit public entities, with history and leadership in the study of ABVs in Mexico. Joint work and multisectoral participation between UADY and CENAPRECE (and other government agencies) has had important effects on public health, generating evidence to design, evaluate, improve, and integrate best practices, new products, and methodologies/technologies for surveillance, prevention, and control of *Aedes* vectors and ABVs, with a regional and global scope. An important interactive and collaborative line of work between UADY-CENAPRECE (through UCBE-UADY) focuses on how to improve surveillance and control of *Ae. aegypti* and ABVs. Since its creation in 2014, UCBE (recognized by the MoH as a collaborative reference center and testing center for the PAHO and WHO) has contributed scientifically to improving surveillance and control of *Ae. aegypti*, a vector of DENV, ZIKV, and CHIKV in Mexico. The UCBE has been included by the WHO as a reference unit for good laboratory practices, as part of a network that aims to generate scientifically reliable data regarding the evaluation of vector control products and methods. Additionally, UCBE colleagues and the LCB team participate on expert committees, working closely with national and/or international governmental and academic institutions.

### 2.6. The Role and Importance of a Social Science Approach

The introduction of new tools such as the SIT-IIT approach requires the involvement of a social science team to understand the targeted communities, potential cultural barriers, perceived social advantages and disadvantages, and eventually, to develop a community engagement strategy for the acceptance and adoption of the biocontrol method. To achieve this goal, we included a multidisciplinary team to explore how the community perceived the mosquitoes (and the diseases that they carry) and whether they viewed them as a problem to be addressed or as a “normal” condition of existence. We engaged with various levels of what are known as “key stakeholders” in the community and broader social groups. The community learned, through theoretical and practical workshops, how to implement good preventive domestic practices to reduce mosquito-breeding sites at home. The introduction of a new paradigm influenced perceptions about both the disadvantages and advantages. Participants also gained insight into how vector control methods work, their limitations, and how innovative approaches can enhance the entire IVM strategy [31].

### 2.7. Collaboration Agreements with Ministries of Health (National, State, and Municipal), as Appropriate to the Country

In February 2016, the Government of Yucatán (GoY) signed an international collaboration agreement with UADY and MSU to develop and apply *Wolbachia*-based strategies to control *Ae. aegypti* and ABVs (DENV, ZIKV, and CHIKV). The GoY ministries involved were the Ministry of Health (SSY), the Ministry of Research, Innovation, and Higher Education (SIIES), and the Yucatán Ministry of Environment (MoE). UADY is also currently updating a collaboration agreement with CENAPRECE for technical support and scaling up WBC for *Ae. aegypti*. Following the successful results of the pilot project [10], the implementation of an IVM model with I*A*M, including releases of male mosquitoes with *Wolbachia* produced with IIT-SIT, is being scaled up in urban areas of Mérida, Yucatán with financial support of the MoH and UADY and a grant from USAID [46]. This is being carried out through a non-profit collaborative model between LCB-UADY, who produce the mosquitoes, and the MoH, who implement the releases in combination with the institutional activities normally included within the program. Currently, there is interest from other states in the Yucatán Peninsula (Campeche and Quintana Roo), with whom a collaboration agreement has been created as part of a national strategic plan led by CENAPRECE.

### 2.8. PAHO Recommendations Through the Regional Program for Public Health Entomology and Vector Control Advisory Group (VCAG)

In 2017, the PAHO created an expert technical group with the purpose of analyzing and evaluating the implementation processes of *Wolbachia*-based projects [47]. This expert group reviewed the advances and results of the WMP initiative for *Ae. aegypti–Wolbachia* population replacement field trials in Medellin, Colombia and Niteroi, Rio de Janeiro, Brazil that provided important insights on the challenges faced by both countries regarding the establishment of the manipulated species into the local environment [48].

## 3. Requirements for the Implementation of the Innovation

After the adoption requirements have been approved, the next stage is to guarantee that other infrastructure, production, technical, and programmatic requirements, as well as social and community acceptance, are available for the implementation (Table 3; see also Table S3 for a summary).

### 3.1. Implementation Plan: Sources of Funding, Long-Term Funding Plan, Input Logistics (Production, Distribution, Release, Monitoring, and Evaluation)

The initiative for the implementation of IIT-SIT through the collaboration of UADY-MoH in Mexico has followed a phased approach based on the developmental roadmap proposed for the testing and deployment of SIT mosquitoes and new vector control technologies [2,49]. The phases are as follows: Phase I: evaluation of biological parameters and efficacy in the laboratory; Phase II: small-scale field pilot trial to assess the entomological efficacy of the intervention, operational feasibility, initial acceptability, and cost; Phase III: larger-scale field trials to assess the efficacy and effectiveness of the intervention (entomological and epidemiological impact); Phase IV: implementation studies under real-world conditions (see also Table S3 for a summary).

The first pilot trial based on the *Wolbachia*-suppression approach in Yucatán was funded by the GoY, UADY, and grants from the National Council for Science and Technology (Mexico) through FOMIX-CONACYT (YUC-2017-03-01-556) and USAID (AID-OAA-F-16-00082) to design, build, equip, and start up a laboratory for the mass production of *Ae. aegypti* mosquitoes, aiming to improve local capacity for the implementation of innovative strategies to reduce mosquito populations and reduce the incidence of *Aedes*-borne diseases. Built on this successful field trial and the existing mosquito mass-rearing capacity established in Mérida, scaling up our WBC for *Ae. aegypti* in Yucatán is not only logical but also feasible. As a continuation, and part of Phase III–Phase IV, a new project for scaling up IIT-SIT as part of an IVM plan for *Ae*. *aegypti* control by the MoH is currently funded by UADY, the GoY, and USAID (APS-7200AA20APS00013).

In agreement with CENAPRECE, UADY, and local MoHs, a non-profit collaboration model has been agreed with LCB-UADY for the first regional mosquito production laboratory and reference center. LCB-UADY will initially produce different lines of *Wolbachia*-carrying *Aedes* for the national and state vector programs, as part of a national strategic plan for the implementation of rear-and-release *Aedes* control methods to be integrated as part of IVM-I*A*M in hotspots in DENV-endemic cities. The envisioned model includes the non-profit cooperation and regional collaboration between LCB-UADY with other state-supported laboratories produce and supply *Ae. aegypti* with *Wolbachia* for public/governmental use, aiming to provide universal access for the public good and support of community health. The MoH will lead this initiative and will carry out community engagement, release, monitoring, and integration with other vector control activities.

### 3.2. Mass-Production System: Physical Infrastructure and Quality Control

#### 3.2.1. Mass Production (Insectary)

The LCB-UADY is located at the CCBA in Mérida, Yucatán, Mexico. LCB-UADY has a collaboration agreement with the GoY and CENAPRECE. It is a unique infrastructure in Mexico, Central America, and the Caribbean and is envisioned as a leading laboratory for the development, evaluation, and implementation of innovative strategies based on mass production and release (rear and release) for the control of *Aedes* mosquitoes. It comprises a facility (237-m^2^) divided into different areas: (1) adult rearing; (2) egg storage, embryo development, and hatching area; (3) larval rearing; (4) pupal separation; (5) Q.C. (taxonomy and molecular biology diagnosis); (6) X-ray room; (7) packing; (8) cleaning; and (9) insectary for species preservation. LCB-UADY has an installed capacity to produce 1 million and the potential to produce 5 million sterile *Ae. aegypti* males per week with IIT, SIT, or IIT-SIT for population suppression [10,30]. Male and female adults as well as eggs (>20 million) can be produced for population replacement with *Ae. aegypti* mosquitoes with *Wolbachia* and low vector competence to DENV, ZIKV, and CHIKV. Currently, various lines of *Ae. aegypti* with *Wolbachia* from the Yucatán Peninsula are established at the LCB.

The vision of the implementation and use of WBC in Mexico is to guarantee universal access to this technology as part of the IVM for the control of *Aedes* and to increase capacities of the programs in the Mexican states. The production model led by CENAPRECE and state MoH envisaged in Mexico is that of small–medium-sized standardized production units (bio-factories) based on the LCB-UADY model. These will be constructed in a stepped manner and distributed across regions/states to address the specific demands for mosquito production in accordance with a national strategic plan for WBC. These mosquito bio-factories can feasibly be built locally with the resources available and will not require major investment in construction and maintenance (e.g., salaries, electricity, etc.). This approach allows manageable administration and Q.C. operations and a capacity production according to current needs. Indeed, these bio-factories could be expanded in a cost-effective manner, depending on expansion and scaling-up plans. Even in the case of automation, this can be developed at a lower cost in these production systems. LCB-UADY has detailed architectural plans and SOPs for the mass production and quality control procedures [50]; these are available with open access and are fully accessible and transferrable to the national and local MoHs.

#### 3.2.2. Quality Control (Laboratory)

Basic quality control processes for the mass production of *Ae. aegypti* within IIT-SIT systems involve the determination, quantification, confirmation, and assurance of parameters such as female contamination rate, pupal size, *Wolbachia* infection in male *Ae. aegypti* mosquitoes, cytoplasmic incompatibility, female sterility after irradiation, and male competitiveness. All these parameters have been reported as initial requirements for the pilot project [10]. The methodology followed at LCB-UADY is available in the handbook of the SOPs for mass production and quality control [42].

### 3.3. Integration with the Local Vector Control Program

#### 3.3.1. IVM Strategy Incorporating IIT-SIT into Routine Control Activities at the MoH

The proposed approach for use of IIT-SIT for population suppression of *Ae. aegypti* in Mexico is as a component of an IVM-I*A*M plan, incorporating preventive field releases of sterile males into routine vector control activities. The generalized tactic and details of how IIT-SIT was introduced and integrated within an IVM plan run by the Mexican MoH has been described previously [10,30].

#### 3.3.2. Criteria Established for Coverage, Frequency, and Volume of Mosquitoes to Be Released

The criteria for coverage, volume, and frequency (dose) of mosquitoes to be released was defined after the pilot field study in Yucatán, based on suburban areas of 30–50 ha [10,30]. For the complete coverage and successful control of such area units, it was decided to conduct mosquito releases twice a week, keeping a ratio of 10:1 between *w*AlbB males and wild-type males, based on the peak of *Ae. aegypti* abundance during the rainy season. Based on prior studies [10,30], we estimated a constant dose of 4000 male mosquitoes per hectare per week, split into two releases of 2000 male mosquitoes per hectare. This number represented a ratio of 10:1 *Wolbachia* to wild-type males, estimated at the peak of the mosquito season. This led us to an estimate of 50 × 4000 = 200,000 males per 50-hectare area unit per week.

#### 3.3.3. Trained Technical Personnel Associated with the Vector Control Program

Implementation of these innovations requires trained personnel from the MoH and control programs to guarantee sustainability of the innovation at the local level. For example, well-trained and experienced field staff from the MoH (three staff vector brigades) and the Collaborative Unit for Entomological Bioassays (UCBE-UADY) (a staff of 10 including one responsible field in charge) successfully monitored *Ae. aegypti* populations and male releases in 50 ha areas during the pilot study in Yucatán [10].

As part of the collaboration with the MoH of Mexico, UCBE-LCB-UADY will be providing continuing education courses in vector control methods and innovative technologies for MoH staff, including *Wolbachia* biocontrol. It is very important to update the staff from vector control programs on other topics, such as the state of the art of implementation of “traditional” methods of surveillance and control, the recommended innovative methods and strategies, risk stratification, and the hotspots approach, among other methods.

#### 3.3.4. Definition of Criteria for Selection of the Areas of Intervention

The selection of control and intervention sites for pilot or initial implementation studies [18] based on the combined traditional–*Wolbachia* IIT-SIT control strategy must be made in consultation with local MoH authorities, considering suitable criteria such as (a) geographically isolation (preferably isolated by local vegetation or natural barriers at least 3 km), (b) areas covering 30–50 ha, (c) preferably suburban areas sharing similar ecological environments for *Ae. aegypti* and socio-demographic conditions (i.e., human population density, rainy season, temperature, housing, services, and infrastructure), and (d) no outbreak or active arbovirus transmission [30].

The Mexican MoH has been exploring a targeted approach for IVM using risk stratification after the identification of high-risk localities, resulting in >100 cities with persistent transmission of ABVs, distributed throughout the country [51]. However, it is evidently impossible for vector control programs in Mexico (or anywhere else) to implement high-quality activities with full coverage in cities (with populations >250,000–1 million inhabitants) where *Ae. aegypti* and ABVs are endemic [52]. To address the challenge of how to make the use of resources more efficient, the MoH of Mexico is currently implementing the identification of hotspots of ABVs as a central element of its national strategy for intra-city stratification [52]. The use of such a framework focusing on ABV transmission risk has been recognized and recommended [22]; therefore, the Mexican model of an IVM-I*A*M program using IIT-SIT will utilize the hotspots approach as guidance for its implementation in urban areas where *Ae. aegypti* and ABVs are endemic.

#### 3.3.5. Costs Associated with the Implementation of New Technologies

Programs based on new technologies are planned for the long term and include mass production and sustained release of sterile male mosquitoes alongside continuous monitoring. The success and sustainability of these programs depend on governmental commitment to pilot trials and scaling up IIT-SIT as part of the national or regional institutional I*A*M strategy. This requires long-term investment in financial resources and trained personnel. Another significant cost associated with these new technologies is the construction of biofactories equipped with standardized mass production systems. Additional expenses include materials and supplies for continuous release, monitoring of entomological indicators, and the subsequent evaluation of their epidemiological impact [18].

The average estimated cost of producing irradiated or *Wolbachia*-carrying mosquitoes is approximately USD 813, assuming a release ratio of 10 mosquitoes per person. For example, Australia demonstrated significant cost variability depending on the area covered (in km^2^), the personnel required (for mass production and field operations), and the duration of release campaigns [18]. To better understand the costs associated with production, release, monitoring, and scaling up mosquito-release interventions within MoH institutional programs, we compared baseline costs for implementing IIT-SIT against those for standard truck-mounted ultra-low-volume (ULV) spraying. This analysis considered expenses for materials, personnel, vehicles, equipment fuel, and consumables for a 50-hectare area. The cost assessment adopted the perspective of the funding agency or end user—in this case, the government—and focused exclusively on direct costs.

The cost of a single application of the insecticide Malathion, approved by the MoH (CENAPRECE), for ultra-low-volume (ULV) spraying over a 50-hectare area is USD 825 per week. Additional operational costs incurred by MoH personnel add USD 38.82, resulting in a total weekly cost of USD 863.82. In contrast, producing male mosquitoes carrying *Wolbachia* for release, assuming a biofactory is already in place, costs USD 340 per week to produce 4000 *Ae. aegypti* males (2000 per hectare, released twice weekly). Field implementation costs—including personnel, fuel, and consumables—amount to USD 63.77 USD, resulting in a total weekly cost of USD 403.77 [10].

Although technological innovations are initially expected to involve substantial costs, each country should adapt the model to leverage existing infrastructure, specialized personnel, and political commitment. Furthermore, it is essential to incorporate these additional costs into existing vector control programs. Since these interventions are complementary and not a substitute for traditional control measures, it is crucial to generate integrated epidemiological, entomological, and economic data. Such information will help define the models and scenarios where IIT-SIT or *Wolbachia*-based strategies can be implemented, scaled up, monitored, and evaluated effectively [18].

### 3.4. Entomological and Epidemiological Surveillance Systems (Ability to Monitor Spatial, Temporal, and Impact Changes)

#### 3.4.1. Entomological Surveillance Capacities Available

Mexico has one of the strongest entomological surveillance systems for *Ae. aegypti* and ABVs in the Americas. Each of the 32 states in Mexico has a vector control program with surveillance and control actions developed according to national regulations and an established work plan. Currently, an extensive network of >200,000 ovitraps is used for entomological surveillance of *Aedes* vectors, which extends to 370 municipalities and 712 urban centers (DENV-endemic/high-risk localities) across the nation. The system can detect *Ae. aegypti* and *Ae. albopictus* mosquitoes and can be used to measure the impact of interventions (i.e., reductions after ultra-low-volume spraying) [53]. One innovation of Mexico’s vector control program is the use of entomological information from ovitrapping for decision making and action, and the occurrence of high/extreme egg counts in ovitraps automatically generates a control response.

A GIS platform, the Integrated Vector Monitoring System (SIMV) [51] recovers and concentrates the information about “egg collections” to calculate primary measures of the presence and abundance of *Ae. aegypti* (egg presence/counts), along with all the entomological information from the programs in all the Mexican states [51]. This platform includes individual sub-systems of information for the surveillance of DENV and urban arboviruses (with surveillance data and recorded activities after vector control interventions, i.e., ULV indoor and outdoor spraying, larval control, etc.), malaria, Chagas disease, and leishmaniasis, including a module on the surveillance of medically important species as well as modules on health promotion, operational procedures, insecticide administration, personnel, vehicles, and application equipment. In particular, the surveillance of DENV and urban arboviruses includes modules of entomological surveillance and comprehensive vector control, entomo–virological surveillance (detection and typing of viruses in mosquitoes collected in the field), evaluation of biological efficacy and resistance to insecticides, and the early warning and response system.

The Mexican MoH entomological surveillance system relies primarily on a large and already established network of ovitraps in the main cities where *Ae. aegypti* and ABVs are endemic, across the whole country. Ovitraps represent an economic and simple method for monitoring *Aedes* populations and can be used as infrastructure to monitor egg hatch rates after release of *Aedes* with *Wolbachia*. Local staff from vector control programs check the ovitraps on a weekly basis (positivity and number of eggs per trap) and input individual Dengue-GIS results into jurisdictional administrative offices.

Adult mosquito collections are not systematically carried out as part of routine activities, although “entomo–virological surveillance” (adult collections and arbovirus detection) is currently being conducted in a few Mexican states, with portable aspirators such as Prokopack aspirators to determine the prevalence and abundance of female mosquitoes and infection with arboviruses [10]. Therefore, indoor adult female collections, which represent the epidemiologically important target, can be more feasible to implement than outdoor collections (with BG traps or others).

#### 3.4.2. Epidemiological Surveillance Capacities Available

The National Epidemiological Surveillance System (SINAVE) from the MoH is a robust system for epidemiological surveillance in Mexico [54]. Additionally, the quality control and technological development for the surveillance are under the direction of the Institute of Epidemiological Diagnosis and Reference (InDRE). Each Mexican state operates a Laboratory of Public Health and Epidemiological Reference for the local diagnosis of infectious diseases. It is mandatory to report DENV, ZIKV, and CHIKV cases, and the system is fed by a network of medical units (hospitals) and health jurisdictions; >90% of all DENV cases (probable cases definition) are geo-referenced in the system. The occurrence of potential/confirmed DENV, ZIKV, and CHIKV cases generates a control response. Rapid, automated exchanges of data on mosquito populations, DENV cases, and control activities ensure effective coordination between the entomological and epidemiological teams.

Entomological data integrated with case reports within SIMV has been used to generate risk maps based on a “transmission risk” index qualitatively subdivided into four categories (low, medium, high, and extreme) and utilized for targeted control. Capitalizing on this impressive data infrastructure, since 2015, the Mexican MoH has applied a simple and robust methodology to identify *Aedes*-borne disease hotspots. These hotspots provide a foundation for risk stratification and the development of strategies for targeted delivery of vector control intervention, representing one of the current examples recommended by the PAHO for risk stratification to guide control programs in the Americas [52,55].

#### 3.4.3. Baseline Situation Assessment (Entomological and Epidemiological) in the Area Where the Innovations Will Be Implemented

UCBE has carried out several evaluations of vector control interventions in Mexico, where *Ae. aegypti* populations have been monitored according to different population parameters (presence, abundance, longevity, etc.) as well as their rates of infection with arboviruses (entomo–virological surveillance), to quantify the impact of interventions. Different interventions can be oriented to target different life stages of the mosquito life cycle; but female mosquito collection (including blood-fed, infected, and particularly indoors, representing the most important epidemiological target) is the “gold standard” for interventions targeting adult vectors, including population suppression strategies such as IIT-SIT [10,27,28]. The collaborative alliance of UCBE-LCB with the Regional Biomedical Research Center (CIR) of UADY oversees the virological and biomedical aspects, including infectious disease epidemiology, social scientific research, and scientific production [26,56].

We conducted a baseline entomological characterization of the abundance and seasonality of *Ae. aegypti* in Yucatán, using ovitraps, BG-sentinel traps and Prokopack aspirators, as part of the pilot study and prior to the release of males with *Wolbachia* produced via IIT-SIT. Information on the seasonal abundance and dynamics of *Ae. aegypti* populations was used to calculate the release ratios and best timing for the releases, as part of an IVM approach, and is being used as a guide for current scaling up in Mérida, Yucatán. The entomological surveillance model for the implementation of IIT-SIT by the MoH includes two phases: (1) baseline pre-release entomological surveys, focused on monitoring *Ae. aegypti* eggs and adults, which are relevant life stages for entomological surveillance of IIT-SIT [4,10,16,30,57,58], and (2) post-release monitoring (cross-sectional collections) of the entomological efficacy (suppression of wild populations of *Ae. aegypti*) using sentinel stations within one-hectare areas [10,30]. Collections with ovitraps are employed to detect/measure oviposition in the peridomicilary areas to determine (i) hatching rates and (ii) the average number of eggs/ovitrap; BG traps with an octenol-based attractant (Octenol Mosquito Magnet) [30] and a sonic attractant from a MAST (male *Aedes* sound trap) [59] are used to calculate peridomestic *Ae. aegypti* density; and Prokopack aspirators are applied for indoor adult mosquitoes [60]. We also monitor fecundity, fertility, parity, survival, competitiveness, longevity, body size, and the emerging of male and female mosquitoes as part of the Q.C. (for more details, see [10,42,50]).

Baseline studies to describe the abundance and phenology of *Ae. aegypti* populations are an initial requirement prior to developing field pilot tests of rear-and-release interventions at any potential study site [2,22,61]. In this context, mosquito surveillance is an essential requisite for monitoring and evaluating future mosquito releases. Two entomological surveillance methods have been commonly performed for assessment of population suppression: the deployment of oviposition traps to monitor the abundance of populations and effective detection of *Wolbachia*-induced CI eggs, and the use of BG-sentinel traps to estimate the infestation levels of adult *Ae. aegypti* [2,4].

Many surveillance tools have been used to calculate the immature stage of the vector mosquitoes. Following several years, the WHO acknowledged that the traditional *Aedes* indices were inadequate for the measurement of DENV vector abundance [62]. Female *Ae. aegypti* is one of the most reliable and direct indicators of DENV exposure [63]. Additionally, it is necessary to conduct routine screening for DENV in adult female *Ae. aegypti* as part of a surveillance program [64]. The collection of female *Ae. aegypti* is a “gold standard” and is desirable for the quantification of mosquito vector populations as a measure of entomological risk (adult females transmit viruses, particularly indoors, because they are highly endophilic), and it is necessary for guidance and the evaluation of methods focusing on reducing/suppressing adult mosquito populations, as is the case with the IIT-SIT. Therefore, the MoH of Mexico has proposed the development of entomo–virological surveillance as part of the program defined as “the search and collection of *Aedes* adults, in DENV endemic and non-endemic localities, with the objective of detecting and identifying circulating serotypes of DENV and/or identifying the presence of new viruses, to establish timely control and prevention strategies”. Although indoor adult collections in Mexico are not yet systematically conducted as part of routine activities, they may be performed with portable devices such as Prokopack aspirators to determine the prevalence and abundance of female mosquitoes and their infection with DENV, CHIKV, and ZIKV [60,65,66,67].

States in Mexico considering the implementation of IIT-SIT will have to augment the capabilities of its entomological surveillance system. Ovitraps are already in use by the Mexican MoH, but adult surveillance will have to be established and performed systematically. Indoor collection of adult females, which represent the epidemiologically important target, has been considered more feasible for implementation by the MoH than outdoor collection (with BG traps).

### 3.5. Principles for Community Engagement and Education: Conceptual Framework and Procedures (Table S3)

At the core of implementing new technology such as a combined IIT-SIT method is the social and cultural inclusion of the communities involved. To achieve this goal, the principles established by the CDC for community engagement are as follows:

1. Respect for cultural diversity: emphasize and include indigenous cultural backgrounds and traditions in the approaches, educative materials, and social branding of the project;
2. Local self-autonomy and social inclusion: enable community-driven projects through the support of local leaders and different social groups by ensuring local involvement and decision making in the implementation stages;
3. Family empowerment: strengthen families’ capabilities, particularly in the context of healthcare, with a focus on mosquito vector control practices;
4. Local appropriation: foster a sense of belonging and commitment among local inhabitants towards the project’s success and sustainability.

#### 3.5.1. Local Participation Agreements with Communities Involved (Informed Consent), as Appropriate to the Country

During the process of social engagement when implementing the entire project in Yucatán, agreements were established with commitments from local leaders and the community families [10,31]. First, meetings were held with the formal and informal leaders and decision makers to present the goals of the intervention, the stages, activities in/outdoors, and the commitments required from them as the main authorities. Once this core group gave the social license for the project, household visits took place to invite families, present the project, describe the participation required, and obtain the signed consent of participants if they agreed to take part in one or more activities. Both phases were conducted in Spanish and the Mayan language (with the support of a local Mayan interpreter).

#### 3.5.2. Perception of Risk or Protection with New Species

In our baseline research, we were able to characterize the culture of participation by participant families regarding health projects implemented in the past [10,31,32]. According to these findings, we designed a participatory approach that considered the cultural particularities of the families.

During the initial fieldwork and further evaluations at the end of the project, perceptions of possible harm to local species was a topic addressed [31,32]. Although most of the adult population in the localities had jobs in the construction or service industries, elders still worked as peasants in the fields. This specific group had concerns about the potential environmental risk of the “new mosquito” released in the intervention locations; they were concerned that the lab mosquitoes could affect their crops, bees, and even livestock [31]. Narratives of old plagues told to the scientific team indicated the reason they reacted in this way. However, the social science team explained to them that this new method was safe and similar to interventions developed in other places in the world. To guarantee the social and linguistic understanding of the scientific terms, we had the support of a local Mayan interpreter that translated all the explanations into the Mayan language.

#### 3.5.3. Perception of Risk of Diseases

Studies about risk perceptions in relation to mosquito-borne diseases were carried out in the community in 2017 as part of the initial rapid diagnosis to identify cultural barriers and opportunities [31,32]. Further evaluations were conducted at the end of the project to determine whether there had been any changes among perceptions over the timeline. The main conclusion was that the participants had higher awareness of the severity of diseases such as DENV, CHIKV, and ZIKV; however, this was not reflected in the adoption of preventive practices to avoid mosquitoes outdoors or inside houses. Here, the increase of “know how” was an important commitment by the team in support of the health education activities related to vector-borne diseases in the community [32]. In addition, participants recommended the implementation of this IIT-SIT approach as part of the scaling up of IVM in other locations in Yucatán.

#### 3.5.4. Strategies for Recruitment and Social–Cultural Inclusion

Specific strategies for recruitment for the *Wolbachia* intervention were implemented with a strong community component, as outlined below.

To increase acceptance levels among participant families, educational demonstration known as “hand in cage” were used to facilitate the community adoption and trust. The activity consisted of participants from the community inserting their hand into a breeding cage containing male mosquitoes with *Wolbachia*. The objective was to demonstrate, interactively, that male mosquitoes do not bite or cause dermatological discomfort and therefore, do not transmit diseases.

Specific promotional and motivational activities implemented in the community for the adoption of the method were as follows:

1. Workshops with children, adolescents, and adults;
2. Discussion groups with local authorities;
3. Scientific tours of the project facilities where the mass production of sterile male mosquitoes was carried out (LCB);
4. Posters about information on the different stages of the project as well as the biological cycle of the mosquito, activities at the LCB, and release of sterile male mosquitoes;
5. Street advertisements to promote the project and invite people to participate in the different activities;
6. Home visit activities allowed sharing of project information, exploring perceptions and its acceptance by the participating and non-participating population.

The indigenous cultural background of the release site promoted a cross-cultural approach [31]. In pursuit of shifting the paradigm “from mosquito eradication to mosquito release”, there was a recognized need to adapt the scientific terminology of IIT-SIT combined methods to a more culturally appropriate language. Meetings were convened to explore the most culturally sensitive and effective approach to achieve this goal; most participants, including elders and community leaders, endorsed the idea of utilizing the Mayan language to convey the project’s key messages and objectives. Through extensive deliberations on challenges and advantages, they conceived the term “Uts k’oxol” (translation: uts = good; k’oxol = mosquito) in the Mayan language as a culturally sensitive branding. Notably, this milestone signified the collaborative effort between the team and local leaders in creating a new word, situated within the indigenous worldview, to introduce the IIT-SIT strategy in Yucatán, Mexico.

**Figure 1:**
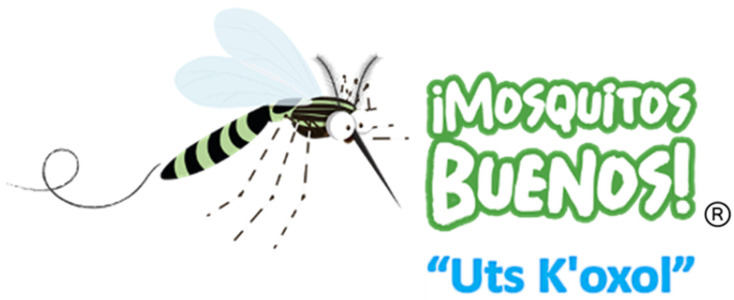
The project´s slogan “Mosquitos buenos and Uts k’oxol”, which translates to “good mosquitoes” from Spanish and Mayan, respectively.

Thus, the “identity of the project” was an important element promoting the use of mass releases of *Wolbachia*-carrying *Aedes* males (Figure 1), considering the sociocultural context of the study area, which gave a sense of belonging and facilitated adoption of the intervention by the community [10,31,32].

#### 3.5.5. Structured Awareness Raising and Communication Campaign

Acceptance testing and raising awareness among stakeholders is a central element in the introduction of technological innovations for *Ae. aegypti* control, in the context of a long history of education and information campaigns and social participation schemes that have promoted changes (effective or not) in individual and social practices to eliminate, protect, or control the variety of vector breeding sites in the domestic and surrounding environment.

Convincing members of the community that it is a good idea to release mosquitoes to do the “dirty work”, when they have already internalized the opposite view, required an extraordinary process of awareness raising and communication to shift people’s perspectives and turn them into participants and partners. The information and awareness campaign explained the features of the innovations (strengths and weaknesses), the release procedures (areas, dates, etc.), the potential risks and especially, the activities in which the community should intervene or participate.

This awareness-raising campaign began with informing and training the various actors at the various levels (national or federal, state or provincial, municipal and local). This included the communicators responsible for reformulating the health promotion strategy and the portfolio of educational messages to include the benefits of the new vector control approach. This is a fundamental step, especially if innovations are introduced as interventions for *Ae. aegypti* control strategies intended to complement the activities of traditional vector control programs (fumigation and elimination). The population was also given several opportunities to raise concerns and receive responses, a basic step in avoiding the spread of rumors and incorrect information.

#### 3.5.6. Communication of Results to Decision Makers, Personnel, and Communities

As part of the activities, several meetings took place in the participant localities and with different social groups, for the presentation of the results, challenges, and achievements of the project. We divided the process into two main sections.

The presentation of results took place in community meetings. Monthly meetings were scheduled to organize the activities and logistics required, for presentation of results, and to receive feedback from the leaders of the community. In addition, meetings were organized every three months to present results and solve any misperceptions or complaints about the project (the scientific team documented these during every household visit), and, also, feedback was necessary to improve our work in the location.

Presentation of results to government agencies took place as part of the institutional agreements and sponsorships. Results were presented in many different forums to the MoH, CONACYT, and USAID. The presentations addressed topics such as development of research protocols, budget and finances, laboratory reports, introduction to the community (release sites), social and educational activities, and the status of community acceptance.

#### 3.5.7. Measurement of Social Acceptance of the Intervention: Challenges and Opportunities

As part of the requirements for the adoption of the combined IIT-SIT approach, we conducted a rapid social assessment aimed at understanding local knowledge about mosquito-borne diseases and experience with vector control programs, with an exploratory approach to the initial acceptance to the whole project [10,31,32]. From the very beginning, one of the major challenges identified was the change of paradigm from “kill” to “release mosquitoes”, thereby facing a local cultural perception of the deeply rooted relations between mosquitoes and humans. However, the main opportunity here was that the community itself had been involved in many vector control program initiatives due to the previous CHIKV and ZIKV outbreaks, in 2015 and 2016, respectively.

To address people’s perceptions of the benefits of these combined techniques, the evaluation played integral roles in our activities [31]. Overall findings indicated the efficacy of the method in reducing *Ae. aegypti* mosquitoes by 90%. Participants demonstrated an understanding of the distinction between wild mosquitoes and those released as part of the project, as well as the importance of integrated vector management (IVM). A significant portion of the population accepted and supported the project, offering feedback to enhance future mosquito-release efforts. The attainment of social license was pivotal for the intervention’s success and should be incorporated into innovative paradigms for mosquito vector control strategies involving community engagement.

In addition, satisfaction monitoring considered the perspectives of two populations: the communities involved, and health and vector personnel involved in the implementation. In accordance with the theoretical framework of the Health Belief Model [68,69], it is suggested to carry out satisfaction monitoring considering the perceived benefits and barriers as well as the signs for action. For monitoring, quantitative and/or qualitative techniques can be combined based on feasibility and the expertise of the research teams in each specific context.

## 4. Consent Tests: Ethical, Social, and Cultural Considerations

When this type of biotechnological innovation is introduced, there is a risk that it will be assumed that the ethical requirements relate only to research (into the biological product) and that social and cultural considerations are not so important, since human beings are not the direct target of the intervention. However, the investigators involved have ethical responsibilities regarding the processes of application and evaluation and the residents of the areas in which the innovations are introduced.

Obtaining the informed consent of the people who will take part, directly or indirectly, in the implementation of the innovations is a minimum requirement that should be broadened as needed to address the needs of the communities concerned. It is also important to address certain social and cultural requirements of the populations with respect to their perceptions, expectations, and needs, not only for information but also for evidence and assurances that their health and that of their family members will not be affected by the application, monitoring, and evaluation of technological innovations. Although these aspects are included as central elements of the preparation phase, many of these activities will have to be conducted throughout the trial, and they may become even more important as the trial advances and the results begin to be registered.

## 5. Conclusions

According to the WHO’s VCAG, this combined method (IIT-SIT) offers the potential for long-term control of *Ae. aegypti* mosquitoes [70]. The evidence collected from other countries in support of reducing natural populations of *Ae. aegypti* using a combined IIT-SIT technique highlights its value as a complementary approach to be adopted across many countries. Incorporating novel technologies into vector control programs is desirable; however, multiple challenges (technical, operational, social, and financial) must be addressed and adapted to the local context in close collaboration with vector control officers and community members.

This novel technique using IIT-SIT has the potential to be incorporated or adapted to the capacity of the MoH and governments (at local and federal levels). However, it requires an open and thorough evaluation of national and local capacities to undertake such a technical transition, particularly in relation to planning, organization, deployment, monitoring, and assessment of how these innovations are integrated, to complement the regular operation of control programs.

The guidelines provided by the PAHO are a valuable resource for all countries for measuring and evaluating their infrastructure and the personnel and technical capacities available for each control program. The case presented by LCB-UADY illustrates the long-term effort made by UCBE to assemble a technical and scientific team. It reflects strong academic bonds with local, state, and national health authorities, as well as the international scientific community, for the development, implementation, and evaluation of potential innovations for the control of *Ae. aegypti* in Yucatán. These are the kind of conditions that will guarantee the successful adoption, implementation, and scaling up of effective and sustainable innovative techniques designed to prevent transmission and suppress vector populations in the long term.

## Figures and Tables

**Table 1:** Requirements for the adoption of technological innovations for *Ae. aegypti* control according to PAHO guidelines [18].

Infrastructure and Programmatic Requirements	Suppression Strategies (Reduce Vector)	Replacement Strategies (Block Transmission)
History of the use of similar technologies for control of agricultural pests (insect sterilization and others)	Desirable	Optional
Regulatory and legislative framework for the use of biotechnologies in the health field:		
-Environmental -Biosafety -Bioethics	Essential Essential Essential	Essential Essential Essential
Protocols for mass production of modified mosquitoes	Essential	Essential
Portfolio (dossier) of evidence on safety, quality, and efficacy of the product	Essential	Essential
PAHO recommendation through the Regional Program for Public Health Entomology and Vector Control Vector Control Advisory Group (VCAG)	Optional	Optional
Collaboration agreements with ministries of health (national or federal, provincial or state, and municipal), as appropriate to the country	Essential (National)	Essential (National)
Implementation plan		
-Guaranteed sources of funding -Long-term funding plan -Input logistics (production, distribution, release, monitoring, and evaluation)	Essential Desirable Essential	Essential Desirable Essential
Physical infrastructure for production of GMM/BMW -Insectarium -Laboratory (entomological) -Material resources for entomological monitoring -Trained technical personnel associated with the vector control	Desirable Essential Optional Essential Essential	Desirable Desirable Essential Essential Essential
Multidisciplinary scientific support group for vector control personnel (action research)	Desirable	Desirable
Entomological surveillance system (ability to monitor spatial, temporal, and impact changes)	Essential	Essential
Epidemiological surveillance system (ability to monitor spatial, temporal, and impact changes, including diagnostic capacity: serology, PCR, isolation)	Desirable	Essential
Baseline situation assessment (entomological and epidemiological) in the area where the innovations will be implemented	Desirable	Essential
Structured raising of awareness and communication campaign (expected impact messages): -Decision makers -Technical personnel -NGOs (environmental, civil, society groups) -Communications media -Communities (formal and informal community groups)	Essential Yes Yes Yes Desirable Yes	Essential Yes Yes Desirable Desirable Yes
Community participation agreements with communities involved (informed consent), as appropriate to the country	Essential	Essential

**Table 2:** Characteristics of *w*AlbB*-Ae. aegypti* line (*w*MID) for release in Mérida, Yucatán, México.

Characteristic	Description	
Backcrossing source	The *Ae. aegypti* line WB2 was generated through embryonic microinjection with the wild-type *Ae. albopictus* line HOU, which carried a native superinfection of *w*AlbA and *w*AlbB [36].	
Outcrossing method	*Ae. aegypti* females *w*AlbB (WB2) were repeatedly outcrossed with wild-type *Ae. aegypti* of Mérida (MID) males for seven consecutive generations.	
*Wolbachia* infection rate	100% of offspring with *w*AlbB from the *w*AlbB-carrying females x uninfected males.	
Fecundity	Eggs per female; 15 females, 1 per conical centrifuge tube; 88.53 ± 19.16 (s.d.).	
Hatch rate	Percentage of hatched eggs for *w*MID line; *w*AlbB-infected female x *w*AlbB-infected male; 300 eggs per 3 replicates; 99.78 ± 0.38% (s.d.)	
Cytoplasmic incompatibility	Percentage of hatched eggs for *w*MID line; 50 females per 3 cages; uninfected female x *w*AlbB-infected male; 0%	
Maternal transmission	Percentage of offspring with *Wolbachia*; 30 females and 30 males per generation; 100 and 99%, respectively.	
Male competitiveness index (C)	Cross mating 1:1:1 per two replicates; Fried Index (C) = 1.29, residual fertility was 0.43 and induced sterility (IS) = 99.57%.	
Body size	Wing length in mm, 30 males and 30 females; 2.27 ± 0.06 (s.d.) and 2.87 ± 0.13 (s.d.), respectively.	
Insecticide susceptibility	CDC bioassay results: % mortality at the diagnostic dose at diagnosis time and 24 h post-exposure.	
	*w*MID—*Ae. aegypti* carrying *w*AlbB.	MID—*Ae. aegypti* without *Wolbachia*.
	Deltamethrin 10 µg/mL—30 min: 100%; 24 h: 100%; status: susceptible.	Deltamethrin 10 µg/mL—30 min: 100%; 24 h: 89%; status: susceptible.
	Permethrin 10 µg/mL—30 min: 100%; 24 h: 97%; status: susceptible.	Permethrin 10 µg/mL—30 min: 98%; 24 h: 78%; status: susceptible.
	Bendiocarb 12.5 µg/mL—30 min: 100%; 24 h: 100%; status: susceptible.	Bendiocarb 12.5 µg/mL—30 min: 100%; 24 h: 100%; status: susceptible.
	Malathion 50 µg/mL—30 min: 100%; 24 h: 100%; status: susceptible. Pirimiphos-methyl 25 µg/mL—30 min: 100%; 24 h: 100%; status: susceptible.	Malathion 50 µg/mL—30 min: 100%; 24 h: 100%; status: susceptible.Pirimiphos-methyl 25 µg/mL—30 min: 98%; 24 h: 100%; status: susceptible.
Reduced vector competence	Vector competence assays were performed to measure the *w*AlbB-mediated blocking (%) effects on DENV, CHIKV, and ZIKV virus infection in *Ae. aegypti* (midgut/salivary gland) at 7 and 14 days post-infection, using a line with a Mexican genetic background [36].	
	ZIKV LP0210Y17 (Asian lineage) and ZIKV/33164Y17 SG(EHI) (South American lineage)—50% infection rate in midgut (95% infection rate in the wild-type mosquitoes). Salivary glands, partial blocking.	
	DENV Serotype 1—N/D	
	DENV Serotype 2—N/D	
	DENV Serotype 3—N/D	
	DENV Serotype 4—N/D	
	Additional testing proving reduced vector competence has been published elsewhere (e.g., [36,45]).	

**Table 3:** Requirements for the implementation of technological innovations for *Ae. aegypti* control according to PAHO guidelines [18].

Implementation	Suppression Strategies	Replacement Strategies
Community engagement and participation in the design, organization, and monitoring of the innovations (local groups)	Desirable	Desirable
Definition of criteria for selection of the areas of intervention (entomological and epidemiological)	Essential	Essential
Integration with local vector control programs	Essential	Essential
Criteria established for coverage, frequency, and volume of mosquitoes released	Essential	Essential
Entomological surveillance (frequency, coverage, level of detail)	Essential	Optional
Case surveillance (confirmed, hospitalized)	Desirable	Essential
Serological/virological surveillance, PCR, and serotypes	Desirable	Essential
Virological and entomological surveillance (PCR in females), molecular biology (genetic and biological fingerprinting)	Optional	Essential
Traditional entomological surveillance:		
-Larval surveys -Oviposition -Pupae -Adults found in homes	Essential Optional Optional Desirable	Essential Optional Optional Desirable
Specialized entomological monitoring (range of flight, fecundity, fertility, parity, survival, vector competence), in line with local capacities	Optional	Desirable
Monitoring of performance and competitiveness (mosquito fitness)	Essential	Essential
Measurement of entomological impact	Essential	Essential
Measurement of epidemiological impact	Essential	Essential
Monitoring of spread of the innovation (establishment and maintenance)	Essential	Essential
Communication of results to decision makers, personnel, and communities	Essential	Essential
Measurement of community acceptance and satisfaction	Desirable	Desirable

## Data Availability

Please see https://doi.org/10.1371/journal.pntd.0010324 and https://correouady-my.sharepoint.com/:f:/g/personal/abdiel_martin_correo_uady_mx/EpLj1hmETHlEr7qArBGO690BgPxNX1gS6Wi3MOsSwYWaVw?e=5H30qq (accessed on 9 September 2024) for data used in this review.

## Supplementary Materials

The following supporting information can be downloaded at: https://www.mdpi.com/article/10.3390/insects15120987/s1, Table S1: Requirements for the adoption of innovations for *Ae. aegypti* control according to PAHO guidelines (PAHO, 2019a), and the use of combined Incompatible Insect Technique and Sterile Insect Technique (IIT-SIT) in Mexico; Table S2: Requirements for the implementation of innovations for *Ae. aegypti* control according to PAHO guidelines (PAHO, 2019a), and the use of combined Incompatible Insect Technique and Sterile Insect Technique (IIT-SIT) in Mexico; Table S3: A Community-lead approach divided into four phases, each one with key milestones and activities described in the subsequent table [10]. References [71,72,73,74] are additionally cited in the supplementary materials.
