# Supplementary material for: Recommendations for Implementing Innovative Technologies to Control Aedes aegypti: Population Suppression Using a Combination of the Incompatible and Sterile Insect Techniques (IIT-SIT), Based on the Mexican Experience/Initiative
Source: Insects. 2024 Dec 12;15(12):987. doi: 10.3390/insects15120987 (PMC11678087; doi:10.3390/insects15120987)
Supplement: Supplementary file 1 [file insects-15-00987-s001.zip › insects-3331241-supplementary.pdf]

insects

MDPI

Supplementary Materials

Table S1. Requirements for the adoption of innovations for *Aedes aegypti* control according to PAHO´s guidelines [1], and the use of combined Incompatible Insect Technique and Sterile Insect Technique (IIT-SIT) in Mexico.

Initial requirements for the adoption of innovations	The case for IIT-SIT in Mexico
Local experience in the use of similar technologies, including pilot-studies	In 2019, the University of Yucatan (UADY) in collaboration with the national and state MoH implemented an IIT-SIT field trial as part of an IVM plan to suppress *Ae. aegypti* in Yucatan, Mexico [2,3]. Male *Ae. aegypti* with *w*AlbB were released during a 24-week period in a 50-ha suburban area, with a significant reduction (90%) observed on the number of indoor *Ae. aegypti* females per house in the treated area during the peak of abundance (rainy season). Thus, it was demonstrated that inundative releases of males produced locally at the LCB-UADY (Laboratory for Biological Control of *Aedes aegypti*) can significantly reduce natural populations of *Ae. aegypti* when integrated within an IVM plan implemented by the MoH.<br>In 2019, the World Mosquito Program (WMP) in collaboration with state MoH performed a population replacement trial with releases of *Aedes aegypti* with *Wolbachia* (*w*Mel) in Baja California Sur, Mexico [4].<br>The sterile insect technique (SIT) has been used to control screwworm flies and Tephritid fruit flies for more than 30 years.
Compliance with regulatory and legislative framework for the use of biotechnologies (environmental, biosafety, bioethics)	Certificates and permits for the introduction and use of *Ae. aegypti* with *w*AlbB from Secretary of Environment and Natural Resources (SEMARNAT) and the MoH of Yucatan.<br>Environmental review scoping statement carried-out by USAID with a local team in Mexico for the pilot trial integrating an *Ae. aegypti* population suppression approach (with IIT-SIT) with IVM done by the MoH in Yucatan, Mexico [2].<br>Protocol of the pilot project reviewed and approved by a local Review Board; community approval (>95%) and informed consents obtained by people participating in the pilot study.<br>Open field mosquito releases approved by the government and performed by the MoH of Yucatan; future use and integration as part of institutional vector control programs will be done by the MoH and as a National Strategic Plan led by the National Center for Preventive Programs and Disease Control of the Mexican Ministry of Health (CENAPRECE).
Availability and accessibility to information about the technology, v.gr. protocols for mass production, implementation, etc.	Handbook of Standard operating procedures (SOPs) & Q.C. of mass-rearing at LCB-UADY, open access, freely and fully available [5]. Reports on the implementation including distribution, release, monitoring, and evaluation of efficacy published in open access peer reviewed scientific publications [2,3]. Other useful documents for population suppression technologies include those developed by the insect [6].
Portfolio (dossier) of evidence on safety, quality, and efficacy of the product	SOPs & Q.C, Laboratory safety & safety procedures, reports on the efficacy published in open access peer reviewed scientific publications [2,5].<br>Although WHO-TAG has not yet published a TPP for *Aedes aegypti-Wolbachia* for population suppression with IIT and/or IIT-SIT, there is sufficient evidence of the characteristics of *Ae. aegypti* with *w*AlbB (as the one used in Mexico) and it is included in this document [7].
Multidisciplinary scientific support group	Groups of national and international experts in vector surveillance and control appointed by CENAPRECE available in Mexico. Each state may have local expert committees to support vector programs, in coordination with corresponding national committees. For example, a multi-sectorial operational research Committee for the Prevention of Mosquito-Transmitted Diseases available in Yucatan.
Collaboration agreements with the Ministry of Health (National, State, or as appropriate to the country)	Initial collaboration agreement for the pilot study with the MoH of Yucatan, SIIES (Secretary of Research, Innovation and Higher Education) and CENAPRECE.<br>Following the successful results of the pilot project, the implementation of an IVM model with IAM (Integrated *Aedes* Management) including releases of male mosquitoes with *Wolbachia* is being scaled up in urban areas of Mérida, Yucatán with financial support of the MoH and UADY and a grant from USAID and support from CENAPRECE.<br>Currently, there is interest from other states in the Yucatan Peninsula (Campeche and Quintana Roo) with whom a collaboration agreement has been established, and which is part of a National Strategic Plan led by CENAPRECE.

Recommendation from PAHO Public Health Entomology Group and/or WHO Vector Control Vector Control Advisory Group (VCAG)	PAHO/WHO offers technical support for pilot studies of new mosquito control technologies (*Wolbachia* and other similar technologies) [8]. The MoH of Mexico recently requested PAHO support for this Mexican model for implementation of WBC for *Ae. aegypti*. Here we provide an assessment of the level of agreement and compliance of the case of the use of IIT-SIT in Mexico, according to PAHO´s guidelines [1]<br>Evidence of IIT-SIT combined for *Wolbachia*-mediated population suppression of *Ae. aegypti*, particularly after the Singapore successful experiences, has been presented to VCAG for its consideration within the intervention class known as "sterilization agents" [9].

Table S2. Requirements for the implementation of innovations for *Aedes aegypti* control according to PAHO´s guidelines [1], and the use of combined Incompatible Insect Technique and Sterile Insect Technique (IIT-SIT) in Mexico.

**Basic requirements for implementation**	**The case for IIT-SIT in Mexico**
Implementation plan:<br>-Sources of funding<br>-Long-term funding plan<br>-Use case model: (Input logistics: production, distribution, release, monitoring, and evaluation)	Construction of LCB and first pilot trial funded by GoY-UADY with grants awarded by CONACYT (YUC-2017-03-01-556) and USAID (AID-OAA-F-16-00082) through Michigan State University.<br>Continuation and scaling up of the project funded by UADY the GoY and USAID (APS-7200AA20APS00013).<br>Use case model: Non-profit collaboration model as an agreement between LCB-UADY-CENAPRECE- local MoHs, LCB-UADY serving as the first regional mosquito production laboratory and reference center. LCB-UADY will initially produce different lines of *Wolbachia*-carrying *Aedes* for the national and state vector programs as part of a National strategic plan for the implementation of rear & release *Aedes* control methods to be integrated as part of IVM-IAM within hot-spots of dengue endemic cities. The model envisioned includes the non-profit cooperation and collaboration of LCB-UADY and other state-supported laboratories producing/supplying regionally *Ae. aegypti* with *Wolbachia* for public/governmental use, this is, to produce public goods for universal access and in support of community health. The MoH will lead this initiative, and will carry out community engagement, release, monitoring, and its integration with other vector control activities.
Physical infrastructure for production and Quality control:<br>-Mass-production s (Insectary)<br>-Quality control (Laboratory)	The Laboratory for Biological Control of *Aedes aegypti* (LCB) of UADY is a laboratory-mosquito bio-factory unique in Mexico with an in-stalled capacity to produce 1 million and the potential to produce 5 million sterile *Ae. aegypti* males per week with IIT, SIT or IIT-TIS for population suppression [2,3] and > 20 million male and female adults, as well as eggs can be produced for population replacement.<br>All parameters performed by the LCB quality control area; methodology available at the handbook of the SOPs for Mass Production and Quality Control [2,5,10], We evaluated the basic parameters such as: female contamination rate, pupal size, wing size, fecundity, fertility, survival, Cytoplasmic incompatibility, male competitiveness, female sterility after irradiation, flight ability and *Wolbachia* infection in male *Ae. aegypti* mosquitoes. As part of UADY facilities, the Virology Laboratory at the Centro de Investigaciones Regionales (CIR-UADY) is a fully functioning laboratory equipped for routine biochemical and molecular genetic analysis such as a) nucleic acid extraction (e.g. DNA, RNA) from mosquitoes, b) detection and quantification of *Wolbachia* genome in infected mosquitoes, c) quality control of *Wolbachia* infection, and d) detection and handling of RNA for arbovirus processing and analyses [11-13].<br>These facilities and capacities will be used as part of the monitoring and evaluation of quality control at UADY as a reference center and will serve to support the states and the National strategic plan. As the gradual transfer of the innovation is carried out to the different states of Mexico, each of them will rely on their regional insectaries and have large and solid facilities and capacities in their state diagnostic laboratories.
Integration with the local vector control program:<br>-IVM strategy incorporating IIT-SIT to routine control activities of the MoH	Integrated control plan for *Aedes aegypti* [2,3], incorporating preventive field releases of male mosquitoes with *Wolbachia* in a preventive manner starting before the rainy season, and continuing during the peak of abundance of *Ae. aegypti* populations and ABVs transmission season. The *Aedes* vector control program of Yucatan, as in most Mexican states, routinely organizes and implements clean up campaigns for breeding-sites disposal (*descacharrización*

-Criteria established for coverage, frequency, and volume of mosquitoes to be released<br>-Trained technical personnel associated with the vector control program<br>-Definition of criteria for selection of the areas of intervention (entomological and epidemiological)	in Spanish) before the rainy season. Truck-mounted ultra-low volume (ULV) application of adulticides from a list of approved products is also performed in response to increased risk of arbovirus transmission, suggested by mosquito abundance. The program additionally performs indoor space spraying and truck mounted ULV in reaction to reported cases of arboviral disease.<br>For the complete coverage and successful control of 30–50 ha area units, it was decided to conduct mosquito releases twice a week keeping a ratio of 10:1 between *w*AlbB males and wild-type males based on the peak of *Ae. aegypti* abundance during the rainy season.<br>Programs will have to calculate and be aware of the personnel required to attend integrated interventions with WBC for *Aedes*, but it is expected that if carried out preventively, efficient use of human resources can be made when there are few cases and no outbreaks, and furthermore, these activities are expected to reduce the magnitude of outbreaks.<br>As part of the collaboration with the MoH of Mexico, UCBE-LCB-UADY UADY will be providing continuing education courses for MoH staff on *Wolbachia* biocontrol and the risk stratification and hotspots approach, among others.
Entomological & Epidemiological surveillance system (ability to monitor spatial, temporal, coverage, and impact changes):<br>-Entomological surveillance capacities available<br>-Epidemiological surveillance capacities available<br>-Baseline situation assessment (entomological and epidemiological) in the area where the innovation will be implemented	CENAPRECE and SSY strong entomological and epidemiological surveillance systems available.<br>The Mexican MoH entomological surveillance system relies primarily on a large and already established network of ovitraps in the main endemic cities of the whole country. Adult collections are not systematically done as part of routine activities, although "entomo-virological surveillance" (adult collections and arbovirus detection) is currently being conducted with portable aspirators such as Prokopack aspirators in a few Mexican States to determine the prevalence and abundance of female mosquitoes and infection with arboviruses [2]. Therefore, indoor adult female collections, which represent the epidemiologically important target can be more feasible to implement than outdoors collections (with BG traps or others).<br>Report of ABVs cases is mandatory, strong capacities for diagnostics; information available from National System for Epidemiological Surveillance, Institute for the Diagnosis and Epidemiology Reference (INDRE), and 32 State Public Health and Epidemiological Reference Laboratories.<br>In 2017, we conducted a baseline entomological characterization of the abundance and seasonality of *Ae. aegypti* with ovitraps, BG-sentinel traps and Prokopack aspirators as part of the pilot study and prior to the releases of males with *Wolbachia* produced with IIT-SIT in Yucatan. Information on the seasonal abundance and dynamics of *Ae. aegypti* populations was used to calculate the release ratios and best timing for the releases as part of an IVM approach. The entomological surveillance used in the pilot-study, and as a model for future implementation of IIT-SIT in new areas by the MoH, included two phases: 1) Baseline pre-release entomological surveys, focused on monitoring *Ae. aegypti* eggs and adults, relevant life stages for entomological surveillance of IIT-SIT [14-17], and 2) post-release monitoring (cross sectional collections) of the entomological efficacy (suppression of wild populations of *Ae. aegypti*) using sentinel stations within one-hectare areas [2,3]. Collections with ovitraps were employed to detect/measure oviposition in the peridomicile to determine i) hatching rates and ii) the average number of eggs/ovitrap; BG-traps with an octenol-based attractant (Octenol Mosquito Magnet) [2], and a sonic attractant from a MAST (Male *Aedes* Sound Trap) [18] to capture female and male to calculate peridomestic *Ae. aegypti* density; and Prokopack aspirators for indoor adult mosquitoes [19]. We also monitored fecundity, fertility, parity, and survival, competitiveness, longevity, body size, and the emerging of male and female mosquitoes as part of the Q.C. process at LCB-UADY (for more details see [2].
Structured awareness-raising and communication campaign:<br>-Decision-makers<br>-Technical personnel<br>-Communications media<br>-Communities (formal and informal groups)	Awareness campaigns conducted in the community through meetings, home visits, presentations of the project in schools, and dissemination using posters [2,20,21].<br>Decision-makers, technical staff, local media, and communities were included in the project [2,20,21].

Community engagement and participation in the design, organization, and monitoring of the innovations (local groups).	Local authorities were included in the design, organization, and monitoring of the combined method innovation.<br>The community got involved providing access to their homes for entomological monitoring (BG traps, ovitraps), mosquito release [2], and a social assessment about the efficacy of the combined method.
Communication of results to decision-makers, personnel, and communities	Monthly meetings were held in which results were presented to decision makers. In addition, the authorities provided support by disseminating the results of the project in the community. During home visits, families were given information on the intervention results and the participants provided relevant feedback for its implementation.
Community engagement and participation in the design, organization, and monitoring of the innovations (local groups)	Local authorities were included in the design, organization, and monitoring of the innovation.<br>The community got involved providing access to their homes for entomological monitoring (BG traps, ovitraps), and mosquito release [2].
Community participation agreements with communities involved (informed consent)	Social and community participation were key to achieving agreements with both authorities and families in the release-sites [2,20]. All families provided their consent to be included in different stages and activities of the intervention.

Table S3. A Community-lead approach divided into four phases, each one with key milestones and activities described in the following table [2].

Phase	Milestones	Activities
Phase I. Preparation of the community.	i) Identification, contact and building bounds of confidence with community leaders and key stakeholders.<br><br>ii) Communication of the goals of the project within the community through house-to-house visits and culturally appropriate materials v.gr. brochures, posters, promotional-video, and educational documents.	Organization of meetings a year prior to the implementation of the vector control activities (2018) with municipal authorities and a local committee of representatives of the community. Presentation of the objectives, expected outcomes, and developed together a framework of collaboration and agreements.<br>From this phase and forward, we arranged monthly meetings with local leaders (usually on Mondays) to present and discuss the progress of the activities, including both the positive results but also the barriers and challenges identified for the implementation of the project. The project established a mechanism of social communication (via the project team) to answer questions or resolve concerns reported by the residents of the study site.<br>After the engagement with local leaders and in collaboration with the MoH, we performed house-to-house visits (N=305) inviting the whole community to participate in the project. Since the beginning, we identified the project needed to design specific messages to characterize and describe the processes related with mass-releases of male mosquitoes as an IVM strategy for vector control, which were done through demonstration activities in workshops and home visits.<br>The cultural identity of the project was an important factor for communication with the population. Mayan identity is very important in Yucatan. The phrase "Uts koxol" ("good mosquitoes" in Mayan language) was used by the project.

Phase II. Pre-releasing community activities	i) Identification of the strengths and cultural barriers for the implementation of the project through social studies and workshops with key leaders of the community.<br>ii) Enrollment and acceptance of the families willing to participate during all the activities of the project thought house-to-house visits and cultural-sensitive materials such as brochures, posters, promotional-videos, and educational documents.	Quantitative surveys and qualitative interviews were performed to characterize the local context of the community, demographics data, knowledge, and social experience on mosquito-borne diseases, emphasizing the perception towards traditional and new methods to control *Aedes*-vector in the locality.<br>One of the major concerns was to understand the perception of the community and the barriers for mass-releases of male mosquitoes. Such a new technology must be well understood for its successful introduction and sustained control.
Phase III. Releasing activities in the whole site	i) Engagement of participants from the community through educational workshops reinforcing goals, commitments, and benefits of the intervention.<br>ii) Reinforcement of the social license of the leaders and the community for the releasing-mosquito process and design a collaborative plan for this activity.	An educational intervention based on PRECED-PROCEEDE model for community adoption of *Ae. aegypti* control with male mosquito releases was developed. Eleven demonstrative workshops were organized at local government facilities to increase the awareness to prevent mosquito-borne diseases, participatory talks about the life cycle of *Ae. aegypti* and its diseases transmitted to humans, and demonstrative explanations of the intervention, the benefits, and the commitments of the participants.<br>The authorities from the release site gave their full support to perform the study. They played an important role as communicators of the project and active monitors of positive and negative impacts produced.<br>In agreement and consent of householders, we selected strategic releasing spots (one per block through the whole community). Families that accepted to become part of the intervention (release points), were in-depth informed about the releasing-schedule and provided with extra educational material to reinforce the knowledge of the intervention, and they became local promoters of the benefits and achievements of the project.
Phase IV. Post-releasing follow-up activities	i) Follow-up/educational activities to reinforce goals, commitments and benefits of the intervention such as house-to-house visits, community meetings, scientific tours to the "mosquito factory".<br><br>ii) Anthropological assessment of the perceived benefits of the project and recommendations for further scaling-up initiatives.	A set of participatory workshops and educative activities were performed at schools addressing the same topics but adapted to scholar populations: 1 puppet theater at the kinder garden, 6 educative workshops at the elementary and 5 educative workshops and mosquito-releasing demonstrations at the secondary levels. In addition, a scientific tour to the "mosquito factory" was organized where students and professors learnt more about the whole mosquito mass production processes.<br>An anthropological assessment was conducted to address the benefits perceived by the community and its local leaders about the IVM project that include traditional and innovative strategies such as the implementation of biological control of *Ae. aegypti* with *Wolbachia*.<br>Finally, press releases were published by local and international newspapers promoting the project as an important Integrated Vector Management to reduce mosquito population in Yucatan, Mexico.

References

1. Pan American Health Organization (PAHO). Evaluation of Innovative Strategies for Aedes aegypti Control: Challenges for their Introduction and Impact Assessment. Washington, D.C. 2019.
2. Martín-Park, A.; Che-Mendoza, A.; Contreras-Perera, Y.; Pérez-Carrillo, S.; Puerta-Guardo, H.; Villegas-Chim, J.; Guillermo-May, G.; Medina-Barreiro, A.; Delfín-González, H.; Méndez-Vales, R.; Vázquez-Narvaez, S.; Palacio-Vargas, J.; Correa-Morales, F.; Ayora-Talavera, G.; Pavía-Ruz, N.; Liang, X.; Fu, P.; Zhang, D.; Wang, X.; Toledo-Romaní, M. E.; Xi, Z.; Vázquez-Prokopec, G.; Manrique-Saide, P. Pilot trial using mass field-releases of sterile males produced with the incompatible and sterile insect techniques as part of integrated *Aedes aegypti* control in Mexico. *PLoS Negl. Trop. Dis* **2022**, *16.*
3. Che-Mendoza, A.; Martin-Park, A.; Chávez-Trava, J. M.; Contreras-Perera, Y.; Delfín-González, H.; González-Olvera, G.; Leirana-Alcocer, J.; Guillermo-May, G., Chan-Espinoza, D.; et al. Abundance and Seasonality of *Aedes aegypti* (Diptera: Culicidae) in Two Suburban Localities of South Mexico, With Implications for *Wolbachia* (Rickettsiales: Rickettsiaceae)-Carrying Male Releases for Population Suppression. *J. Med. Entomol* **2021**, *58*, 1817–1825.
4. World Mosquito Program (WMP), Available online: https://www.worldmosquitoprogram.org/en/global-progress (accessed on 23 July 2024).
5. Contreras-Perera, Y.; Pérez-Carrillo, S.; Martin-Park, A.; Puerta-Guardo, H.; Che-Mendoza, A.; Pavía-Ruz, N.; Manrique-Saide, P. Manual de procedimientos. Sistema de producción masiva de mosquitos *Aedes aegypti* con *Wolbachia*, 1st ed.; Publisher: Shanti Nilaya, Mexico, 2023. ISBN 978-1-961809-02-4.
6. International Atomic Energy Agency. Available online: https://www.iaea.org/topics/insect-pest-control/laboratory.
7. World Health Organization. Available online: https://cdn.who.int/media/docs/default-source/ntds/vector-ecology-mangement/tpp-wolbachia-infected-aedes-aegypti-population-replacement-intervention.pdf?sfvrsn=5296c1dc_3 (accessed 17 September 2023).
8. Pan American Health Organization/World Health Organization. Available online: https://www.paho.org/en/news/8-4-2016-paho-offers-provide-technical-support-pilot-studies-new-mosquito-control-technologies (accessed 27 November 2023).
9. World Health Organization & The International Atomic Energy Agency (WHO/IAEA). *Guidance Framework for Testing the Sterile Insect Technique (SIT) as a Vector Control. Tool against Aedes-Borne Diseases.* Publisher: Geneva: World Health Organization and the International Atomic Energy Agency; 2020. https://iris.who.int/handle/10665/331679.
10. Contreras-Perera, Y.; Flores-Pech, J.P.; Pérez-Carillo, S.; et al. Different larval diets for *Aedes aegypti* (Diptera: Culicidae) under laboratory conditions: in preparation for a mass-rearing system. *Biol* **2023**, *78*, 3387–3399.
11. Puerta-Guardo, H.; H., Contreras-Perera, Y.; Perez-Carrillo, S.; Che-Mendoza, A.; Ayora-Talavera, G.; Vazquez-Prokopec, G.; et al. *Wolbachia* in Native Populations of *Aedes albopictus* (Diptera: Culicidae) From Yucatan Peninsula, Mexico. *J Insect Sci* **2020**, *20*(5):16.
12. Manrique-Saide, P.; Herrera-Bojórquez, J.; Medina-Barreiro, A.; Trujillo-Peña, E.; Villegas-Chim, J.; Valadez-González, N.; Ahmed, A. M. M.; Delfín-González, H.; Palacio-Vargas, J.; Che-Mendoza, A.; Pavía-Ruz, N.; Flores, A. E.; Vazquez-Prokopec, G. Insecticide-treated house screening protects against Zika-infected *Aedes aegypti* in Merida, Mexico. *PLoS Negl. Trop. Dis* **2021**, *15*.
13. Kirstein, O.D.; Ayora-Talavera, G.; Koyoc-Cardeña, E.; Chan-Espinoza, D.; Che-Mendoza, A.; Cohuo-Rodriguez, A.; Granja-Pérez, P.; Puerta-Guardo, H.; Pavia-Ruz, N.; Dunbar, M. W.; Manrique-Saide, P.; Vazquez-Prokopec, G. M. Natural arbovirus infection rate and detectability of indoor female Aedes aegypti from Mérida, Yucatán, Mexico. *PLoS Negl. Trop. Dis* **2021**, *15*, e0008972.
14. Kittayapong, P.; Ninphanomchai, S.; Limohpasmanee, W.; Chansang, C.; Chansang, U.; Mongkalangoon, P. Combined sterile insect technique and incompatible insect technique: The first proof-of-concept to suppress *Aedes aegypti* vector populations in semi-rural settings in Thailand. *PLoS Negl Trop Dis* **2019**, *13*.
15. Mains, J.W.; Brelsfoard, C.L.; Rose, R.I.; Dobson, S.L. Female adult *Aedes albopictus* suppression by *Wolbachia*-infected male mosquitoes. *Scientific reports* **2016**, *6*.
16. Zheng, X.; Zhang, D.; Li, Y.; Yang, C.; Wu, Y.; Liang, X.; Liang, Y.; Pan, X.; Hu, L.; Sun, Q.; et al. Incompatible and sterile insect techniques combined eliminate mosquitoes. *Nature* **2019**, *572*, 56–61.
17. Crawford, J.E.; Clarke, D.W.; Criswell, V.; Desnoyer, M.; Cornel, D.; Deegan, B.; Gong, K.; Hopkins, K.C.; Howell, P.; Hyde, J. S.; et al. Efficient production of male *Wolbachia*-infected *Aedes aegypti* mosquitoes enables large-scale suppression of wild populations. *Nat. Biotechnol* **2020**, *38*, 482–492.
18. Staunton, K.M; Leiva, D.; Cruz, A.; Goi, J.; Arisqueta, C.; Liu, J.; et al. Outcomes from international field trials with Male *Aedes* Sound Traps: Frequency-dependent effectiveness in capturing target species in relation to bycatch abundance. *PLoS Negl Trop Dis* **2021,** *15*(2): e0009061.
19. Vazquez-Prokopec, G.M.; Galvin, W.A.; Kelly, R.; Kitron, U. A new, costeffective, battery-powered aspirator for adult mosquito collections. *J Med Entomol* **2009**, *46*, 1256-1259.
20. Villegas-Chim, J.; Martin-Park, A.; Puerta-Guardo, H.; Toledo-Romaní, M. E.; Pavía-Ruz, N.; Contreras-Perera, Y.; Pérez-Carrillo, S.; Che-Mendoza, A.; Palacio-Vargas J.; Correa-Morales, F.; Gómez-Dantés, H.; Manrique-Saide, P. Community engagement and social assessment for *Wolbachia*-based suppression of natural populations of *Aedes aegypti*: In *The Mexican experience. In Mosquito Research-Recent Advances in Pathogen Interactions, Immunity, and Vector Control Strategies*. Puerta-Guardo H.; Manrique-Saide M. Eds. *IntechOpen*. Publisher: London, United Kingdon, **2022**. ISBN 978-1-80355-853-0.

21. Martinez-Cruz, C.; Arenas-Monreal, L.; Gomez-Dantes, H.; Villegas-Chim, J.; Barrera-Fuentes, G.; Toledo-Romani, M. E.; Pavia-Ruz, N.; Che-Mendoza, A.; Manrique-Saide, P. Educational intervention for the control of *Aedes aegypti* with *Wolbachia* in Yucatan, Mexico. *Eval. Program Plan* **2023**, *97*.
